# Analysis and Multi-Objective Optimization for Reducing Energy Consumption and Improving Surface Quality during Dry Machining of 304 Stainless Steel

**DOI:** 10.3390/ma13214693

**Published:** 2020-10-22

**Authors:** Feilong Du, Lin He, Haisong Huang, Tao Zhou, Jinxing Wu

**Affiliations:** 1 Key Laboratory of Advanced Manufacturing Technology of the Ministry of Education, Guizhou University, Guiyang 550025, China; fldu@gzu.edu.cn (F.D.); hshuang@gzu.edu.cn (H.H.); 2 School of Mechanical Engineering, Guizhou University, Guiyang 550025, China; gz_zhoutao@163.com (T.Z.); gs.pftian17@gzu.edu.cn (J.W.); 3 School of Mining & Civil Engineering, Liupanshui Normal Colleague, Liupanshui 553004, China

**Keywords:** 304 stainless steel, desirability analysis, cutting parameter, specific cutting energy, surface roughness, microhardness, multi-objective optimization

## Abstract

Cutting quality and production cleanliness are main aspects to be considered in the machining process, and determining the optimal cutting parameters is a significant measure to reduce energy consumption and optimize surface quality. In this paper, 304 stainless steel is adopted as the research objective. The regression models of the specific cutting energy, surface roughness, and microhardness are constructed and the inherent influence mechanism between cutting parameters and output responses are analyzed by analysis of variance (ANOVA). The desirability analysis method is introduced to perform the multi-objective optimization for low energy consumption (LEC) mode and low surface roughness (LSR) mode. Optimal combination of process parameters with composite desirability of 0.925 and 0.899 are obtained in such two modes respectively. As indicated by the results of multi-objective genetic algorithm (MOGA), genetic algorithm (GA) combined with weighted-sum-type objective function and experiment, the relative deviation values are within 10%. Moreover, the results also reveal that the feed rate is the most significant factor affecting the three responses, while the correlation of cutting depth is less noticeable. The effect of low feed rate on microhardness is primarily related to the mechanical load caused by extrusion, and the influence at high feed rate is determined by plastic deformation.

## 1. Introduction

Because of heat resistance, corrosion resistance, and other advantages, 304 stainless steel has been commonly used in various industries such as aerospace, construction, automotive, and medical [1]. Nevertheless, 304 stainless steel is identified as a difficult-to-machine material owning to poor surface quality, high cutting force and cutting temperature, and high wear during machining [2]. Therefore, the machining performance and surface quality of stainless steel have always been the focus of attention for research [3,4].

Manufacturing plays a critical role in social development, and sustainable manufacturing is a significant branch of machinery manufacturing. As for machining, it involves various factors having potential for the sustainable development, including cooling and lubricating fluids, energy consumption, waste disposal of fluids, and so on [5]. At present, cutting fluid machining represents the most common machining method, but it has the potential to put the health of the operator and environmental safety at risk [6]. In order to address these adverse effects, many scholars pay attention to the ecological trends in machining processes, especially the impact of different cooling-lubricating techniques on the machining. Krolczyk et al. [7] reported a balance between the improvement of cutting and the reduction to pollution caused by coolants and emulsions, based on which some sustainable manufacturing methods were proposed, including dry cutting, cryogenic, MQL/MQCL, and so on. Considering the current trend, Sen et al. [8] re-examined the development of MQL technology, and further studied the benefits of vegetable oil and nano oil as lubricants. The existing studies demonstrate that the new coolant technologies can produce a satisfactory performance in heat exchange [9], surface integrity, and tribological properties [10]. However, the high cost of lubrication technology, energy consumption, and cutting fluid waste management have contributed to higher manufacturing costs and serious environmental issues. Therefore, dry cutting is considered the most appropriate machining method for sustainable manufacturing whenever possible.

The choice of cutting parameters has an important impact on machining performance [11,12]. However, because of the characteristics of high heat capacity and thermal conductivity for 304 stainless steel, improper selection of process parameters in dry cutting process may lead to not only excessive energy consumption [11] but also deterioration in surface machining quality [12]. Therefore, a correct choice of cutting parameters plays a crucial role in the determination of cutting scheme for 304 stainless steel. Single-objective optimization, as a common approach of cutting parameter analysis, is significant in determining optimal cutting conditions to some extent. Despite this, it is of limited value if multiple different and contradictory objectives are optimized simultaneously [13]. As the machining demands increase, usually two or more objectives need to give consideration. Therefore, only through multi-objective optimization can the optimal cutting parameters be determined effectively.

Currently, a majority of the common cutting parameters are optimized by means such as response surface methodology (RSM), Taguchi test, parameter space investigation (PSI), artificial neural network (ANN) to construct the corresponding objective prediction model and obtain optimal solutions to parameters. Taking Ti-6Al-4V as the research objective, Mia et al. [14] conducted an analysis of the different forces and surface roughness by Taguchi test, based on which a corresponding response model was established with the assistance of RSM and ANN. Despite this, the experimental results were not verified. Aiming at Ti-6Al-4V, Paschoalinoto et al. [15] used ANN to study the influence of cutting parameters on cutting force, torque, and surface roughness and optimized accordingly under different lubrication conditions. Under different cooling conditions, Maruda et al. [16] studied the influence mechanism of cutting parameters and active medium-related parameters on the chip formation zone by parameter space investigation (PSI) method. During the cutting of S50C medium carbon steel, Masmiati et al. [17] predicted the surface roughness, cutting force, and residual stress with the assistance of RSM. Desirability analysis provides a method to evaluate the degree of optimization for a single or a group of responses. This method is widely conducted in satisfaction surveys of various social problems because it converts multi-response values optimization into single-response values optimization with such advantages as simplicity, flexibility, and high explainability. Some scholars have introduced the desirability analysis method into the multi-objective optimization process of cutting [18]. Bhushan et al. [19] optimized the cutting parameters and obtained the minimum power consumption and maximum tool life by the desirability analysis. However, the surface quality was ignored as an optimization objective in the study. Based on the desirability analysis method, Mobin et al. [20] proposed a parameter optimization method of the evolutionary optimization algorithm. They explored multi-objective particle swarm optimization (MOPSO) and non-dominated sorting genetic algorithm (NSGA-III) and identified the optimal parameters of the evolutionary optimization algorithm. Though the performance indicators were optimized, an improvement to the practicability and simplicity of the method remained necessary. Saidi et al. [21] reported the significant cutting parameters that impact on the surface roughness, tangential force, and material removal rate by analysis of variance, and performed optimization from the perspectives of quality, productivity and mass-productivity combination based on the desirability function. The research showed certain significance to the optimization of productivity and surface roughness. However, the impact of energy consumption was excluded from the analysis. In the existing studies, the desirability method has not been used for multi-objective optimization that fully combines energy consumption and surface quality under different cutting conditions. In addition, there is a lack of comparison between the optimization results of desirability method and other optimization methods.

The multi-objective optimization of austenitic stainless steel is also suggested by many researchers [22,23]. Not only does energy consumption make a difference to the machining cost, it is but also an important aspect that must be factored into cleaner production. Some scholars have included energy consumption as an objective for the multi-objective optimization process of austenitic stainless steel cutting. Under dry cutting condition, Bagaber and Yusoff [24] performed turning experiments on 316 stainless steel, and carried out multi-objective optimization of power consumption, tool wear, and surface roughness with the assistance of RSM. As revealed by the results, the three responses were noticeably reduced by 14.94%, 13.98%, and 4.71% respectively. Additionally, the reliability of the results was validated. Aiming at the minimum surface roughness and minimum energy consumption, Muñoz-Escalona et al. [25] conducted an investigation into the milling process of austenitic stainless steel and identified the optimal combination of the relevant parameters.

The majority of current cutting parameter optimization studies concentrate on energy consumption, surface roughness, residual stress, tool wear, and so on, while the research on microhardness remains rare. As an indispensable aspect of surface integrity, microhardness is of great significance throughout the life cycle of a production [26]. Microhardness has an important influence on the yield strength [27], wear resistance of the material, and the bearing capacity of the structure [28]. Therefore, it is practically significant to investigate microhardness. Zhang et al. [29] constructed a multi-physical model and a martensitic transformation model for 304 stainless steel cutting process and made predictions of microhardness and residual stress respectively, in addition to carrying out experiments to validate the correctness of the model. With different cutting parameters, Krolczyk et al. [30] analyzed the microhardness in dry turning of 1.4541 austenitic stainless steel. Sharma et al. [3] researched the relationship between machining parameters and different objective responses for 304 stainless steel and further analyzed the influence of microhardness on tribological properties.

In summary, the existing research on microhardness is basically focused on mechanism analysis and single objective prediction. The multi-objective optimization of microhardness combined with cutting energy and surface roughness is particularly significant for enhancing cutting performance, improving surface machining quality and reducing machining energy consumption, while the current investigation is rarely seen. Therefore, aiming to reduce energy consumption and optimize surface quality, on the one hand, the study establishes prediction models of energy consumption, surface roughness, and microhardness, and explores the influence mechanism of cutting parameters on various objective responses. On the other hand, microhardness is combined with energy consumption and surface roughness, and the method of desirability analysis is introduced to optimize cutting parameters for low energy consumption mode and low surface roughness mode with a higher level of microhardness range.

## 2. Methodology

### 2.1. Desirability Analysis

Desirability analysis is a mathematical method to find the optimum, and the purpose is to obtain a composite desirability value as large as possible to meet favorable conditions of all goals. Based on the characteristics of objective responses, the desirability function includes the maximum goal, the minimum goal, and the in-range goal:

For the goal of maximum, the desirability is defined by Equation (1):

(1) $$d_{i}=\{\begin{array}{c} 0,y_{i}\leq y_{\min}\\ {\left[\frac{y_{i}-y_{\min}}{y_{\max}-y_{\min}}\right]}^{w_{i}},y_{\min}<y_{i}<y_{\max}\\ 1,y_{i}\geq y_{\max} \end{array}$$

In order to achieve the minimum goal, the desirability is defined by Equation (2):

(2) $$d_{i}=\{\begin{array}{c} 1,y_{i}\leq y_{\min}\\ {\left[\frac{y_{\max}-y_{i}}{y_{\max}-y_{\min}}\right]}^{w_{i}},y_{\min}<y_{i}<y_{\max}\\ 0,y_{i}\geq y_{\max} \end{array}$$

For the goal within range, desirability is determined through Equation (3):

(3) $$d_{i}=\{\begin{array}{c} 0,y_{i}\leq y_{\min}\\ 1,y_{\min}<y_{i}<y_{\max}\\ 0,y_{i}\geq y_{\max} \end{array}$$

Composite desirability is calculated by Equation (4):

(4) $$D={\left[\prod_{i=1}^{n}\left(d_{i}^{r_{i}}\right)\right]}^{\frac{1}{\sum r_{i}}}$$

where *d_i_* indicates the individual desirability of *y_i_, y*_max_ denotes the upper limit of *y_i_, y*_min_ refers to the lower limit of *y_i_, w_i_* represents the individual weight of *y_i_, D* indicates the composite desirability, and *r_i_* stands for the individual importance of *y_i_*.

### 2.2. Cutting Energy Theory

In the process of machining, the energy consumed by removing material per unit volume is defined as specific cutting energy, which is determined by Equation (5) [31].

(5) $$N_{s}=\frac{F_{c}\times v_{c}}{MRR}$$

where *F_c_* indicates the main cutting force obtained from the force measuring system, *ν_c_* denotes the cutting speed, and *MRR* represents the material removal rate.

*MRR* is calculated according to Equation (6)

(6) $$MRR=v_{c}\times f\times a_{p}$$

where *f* indicates the feed rate, and *a_p_* denotes the cutting depth.

Calculated from the Equations (5) and (6)

(7) $$N_{s}=\frac{F_{c}}{f\times a_{p}}$$

**Figure 1:**
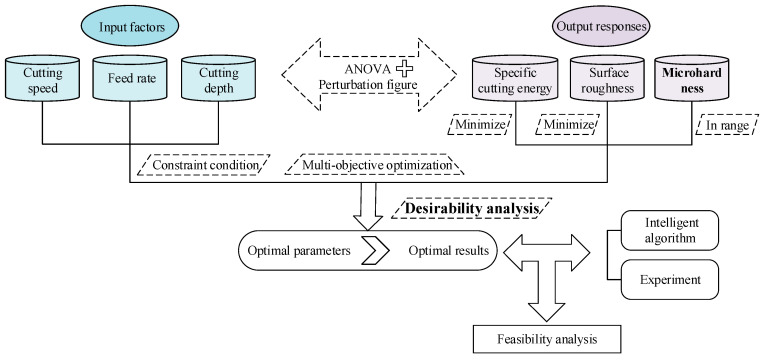
Flow chart of the study.

In this study, the main cutting force (*F_c_*), surface roughness (*R_a_*) and microhardness (*HV*) are obtained by cutting test measurement system and the specific cutting energy (*N_s_*) is calculated by the Equation (7). With the cutting speed (*ν_c_*), feed rate (*f*), and cutting depth (*a_p_*) taken as inputs, the corresponding regression equation is obtained, and the inherent influence mechanism between cutting parameters and output responses is analyzed by ANOVA combined with perturbation diagram. Then, with microhardness as an important response and its value limited to a higher range, the cutting parameters in low energy mode and low surface roughness mode are optimized using the desirability function under the machining constraints. Finally, the effectiveness of multi-objective optimization results is validated by performing intelligent algorithm optimization and experiment. Figure 1 illustrates the flow chart of the study.

## 3. Experimental Design

### 3.1. Workpiece and Turning Inserts

**Figure 2:**
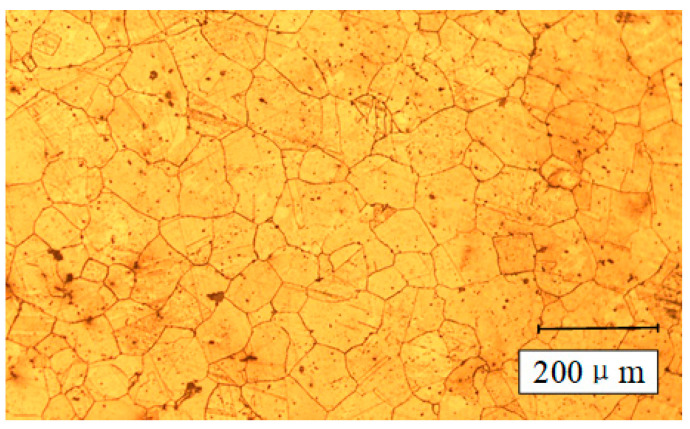
Micrograph of material matrix microstructure.

In the cutting experiment, the workpiece is rod-shaped 304 stainless steel with a diameter of 68 mm and an axial length of 140 mm. In addition, each axial cutting length is set to 10 mm, and the position at 5 mm is selected for measurement. The composition of workpiece material is shown in Table 1, and Figure 2 shows the micrograph of material matrix microstructure. The turning inserts are supplied by a certain manufacturer and insert code is CNMG120404, which are made of tungsten carbide-coated tool intended for difficult-to-machine material, and the coating is 5 μm TiAlN. In addition, the symbol of holder is BCLNR2525M12. The geometric and working angles are listed in Table 2.

### 3.2. Measurement System

**Figure 3:**
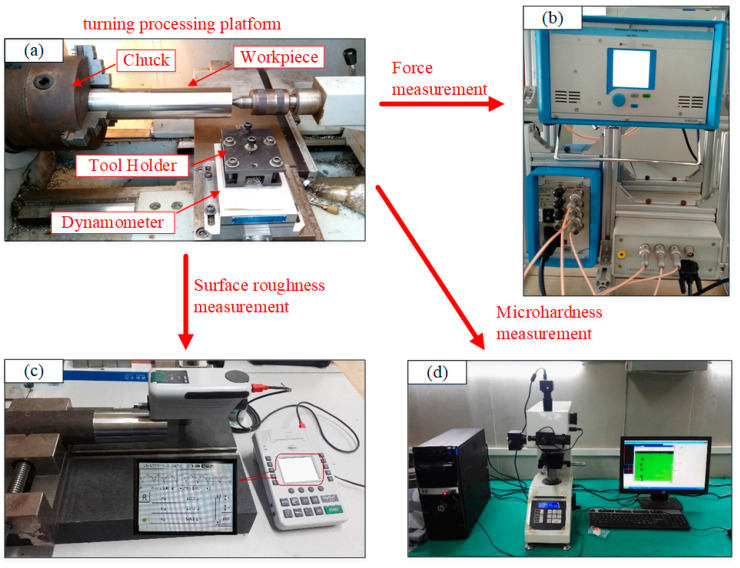
Cutting test measurement system: (**a**) turning processing platform; (**b**) cutting force measurement platform; (**c**) surface roughness measurement platform; (**d**) microhardness measurement platform.

Figure 3 shows a cutting test measurement system. The cutting experiment is conducted with the assistance of the C6136HK CNC lathe. So as to ensure the uniformity of machining throughout the cutting process and mitigate the impact caused by rust or hardening on the workpiece surface, 304 stainless steel is precut with a cutting depth of 1 mm before cutting. The turning processing platform is illustrated in Figure 3a. Kistler force measuring system is installed, and the cutting force measurement platform is shown in Figure 3b. The measurement is carried out for three times under each cutting condition, and the average is taken as the actual measurement value. For the sake of reducing the measurement deviation to the minimum, three different positions in the middle spaced at an angle of 120° are selected for measurement, and the average value is calculated, which is performed by the Mahr benchtop probe roughness detection platform. The surface roughness measurement platform is illustrated in Figure 3c. Microhardness in Vickers (*HV*) is carried out with the assistance of DHV-1000Z digital micro Vickers hardness tester. Figure 3d presents a microhardness measurement platform.

**Figure 4:**
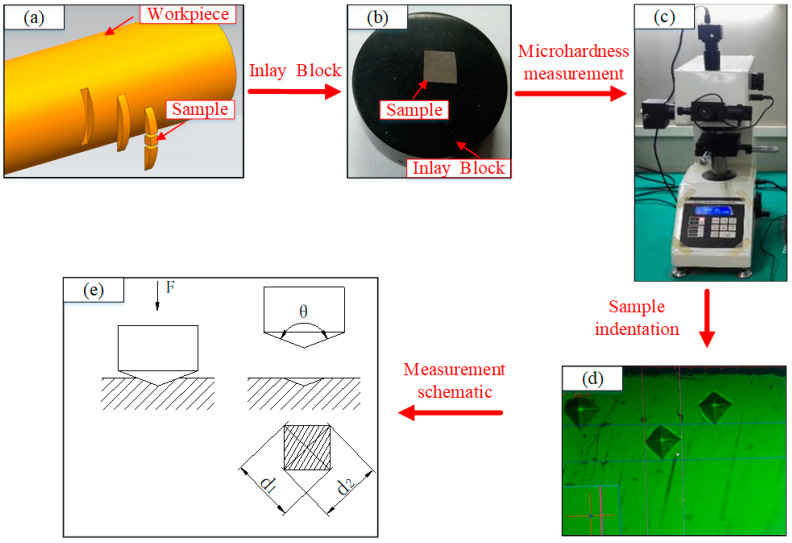
Microhardness measurement process and relevant samples: (**a**) sampling schematic; (**b**) inlay sample; (**c**) microhardness tester; (**d**) sample indentation; (**e**) measurement schematic.

Figure 4 shows the microhardness measurement process and relevant samples. The basic procedures are as follows: first, the 304 stainless steel bar is sampled by wire cutting with sufficient coolant and low cutting speed, and a sample with a size of 5 mm × 5 mm × 5 mm is cut near the middle of the axial and circumferential direction, and Figure 4a shows a sampling schematic. Then, the obtained sample is inlaid, that is, put the side with arc segment face downward, and another side with arc segment is reserved as the test plane. The inlay sample is shown in Figure 4b. Finally, the sample is ground and polished, and the subsurface hardness of the sample is tested by microhardness tester illustrated in Figure 4c. The sample indentation is shown in Figure 4d.

The measurement principle and process of microhardness are as follows: The indenter of the microhardness tester is pressed into the surface of the sample under a fixed force of 0.05 kgf (0.05 HV), after 10 s the test force is removed, and length of the diagonal of the sample indentation is measured as shown in Figure 4e. A number of points are taken to perform measurement at a distance of 25 μm from the machined surface. In order to reduce the error, the sample under each working condition is measured three times and the average is taken as the actual value. The distance between any two measurement points is 30 μm, which is convenient to avoid the mutuality between the test points influences. The quotient obtained by dividing the test force by the sample indentation cone surface area represents the Vickers hardness value. The calculation equation of the hardness value is as follows:

(8) $$HV=\frac{F}{S}=\frac{2F\times \sin\;(\;\theta /2)}{d^{2}}$$

In the equation, *HV* represents the Vickers hardness value, *F* indicates the test force, *S* is the area of indentation cone surface, *d* refers to the average value of the two diagonal lengths (*d*_1_ and *d*_2_) of sample indentation, *θ* represents the angle between the two opposite sides of the indenter.

### 3.3. Design of Experiments

#### 3.3.1. Cutting Parameters

According to the tool type and the cutting parameters selected by manufacturer, a three-level design is adopted for each cutting parameter, which is represented by “−1,” “0,” and “1” respectively. The experimental factors and levels are indicated in Table 3. The upper limit and lower limit of each parameter are determined according to the recommendation from the manufacturer, while the value of Level 2 is defined as the average of the maximum and the minimum.

#### 3.3.2. Experimental Design and Calculation

Compared with the partial and quarter factorial experiment, the full factorial design can not only identify the main effect factor, but also explore the relationship between interaction items and responses comprehensively, thereby establishing a more accurate regression model. Therefore, a three-level design for three factors (3^3^ = 27) is designed and experiments of 27 samples are conducted. The values of specific cutting energy, surface roughness, and microhardness under different parameter combinations are obtained. In Table 4, the design and results of the experiment are revealed.

## 4. Results and Discussion

### 4.1. Analysis of Variance (ANOVA)

The correlation coefficient can be used to measure the trend of two variables changing simultaneously. In this study, Pearson product moment correlation is adopted to study the relationship between different cutting parameters and output responses. The correlation between factors and responses is indicated in Table 5. As for surface roughness and specific cutting energy, feed rate is shown to be the most relevant factor, with the correlation value being in excess of 0.7. The correlation is weak between cutting depth and surface roughness (−0.158) and specific cutting energy (−0.081). As for microhardness, feed rate and cutting speed are identified as significant factors, with the correlation value being invariably more than 0.5.

The analysis of variance (ANOVA) is used to analyze the relationship between factors and responses in a more effective and accurate way. Table 6, Table 7 and Table 8 present the ANOVA data of specific cutting energy, surface roughness, and microhardness, respectively.

As indicated by Table 6, Table 7 and Table 8, the F-values of three responses are 151.47, 121.09, and 98.12 respectively, which suggest that the models are significant. There is merely a 0.01% chance that a “Model F-Value” such large could occur because of noise. As the values of Pred R-Sq (Predicted multiple correlation coefficient), R-sq (Multiple correlation coefficient), and Adj R-Sq (Adjusted multiple correlation coefficient) deviate from 1 very little, the three response models are not overfitting and demonstrate sufficient predictability. So these models are reliable. 

### 4.2. Establishment and Analysis of Regression Equation

#### 4.2.1. Specific Cutting Energy (*N_s_*)

According to the analysis of variance, the model items with correlations that are inconsistent with the requirements are removed, and the second-order regression prediction model is obtained.

(9) $$\begin{array}{cc} N_{s}= & 3267-11.34\times v_{c}-27252\times f+3142\times a_{p}+\;0.02656\times \\ & v_{c}{}^{2}+\;79569\times f^{2}-1287\times a_{p}{}^{2}+2993\times f\times a_{p} \end{array}$$

**Figure 5:**
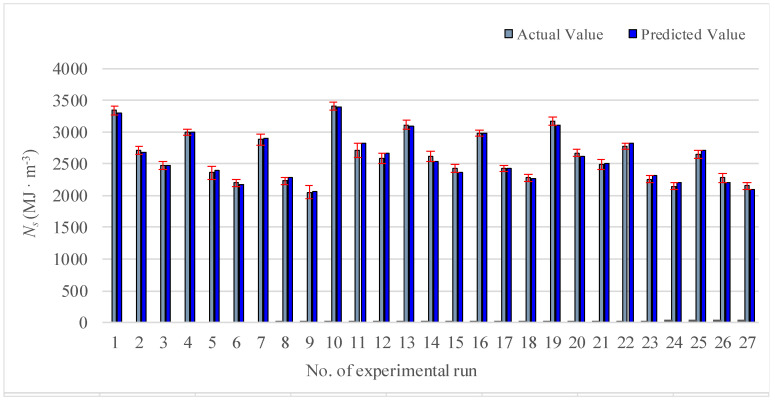
Comparison chart between predicted value and measured value (N_s_).

By analyzing the regression model of Equation (9), the value comparison performed between the prediction and the measurement is shown in Figure 5. The predicted value is close to the actual value, indicating the accuracy of the model.

**Figure 6:**
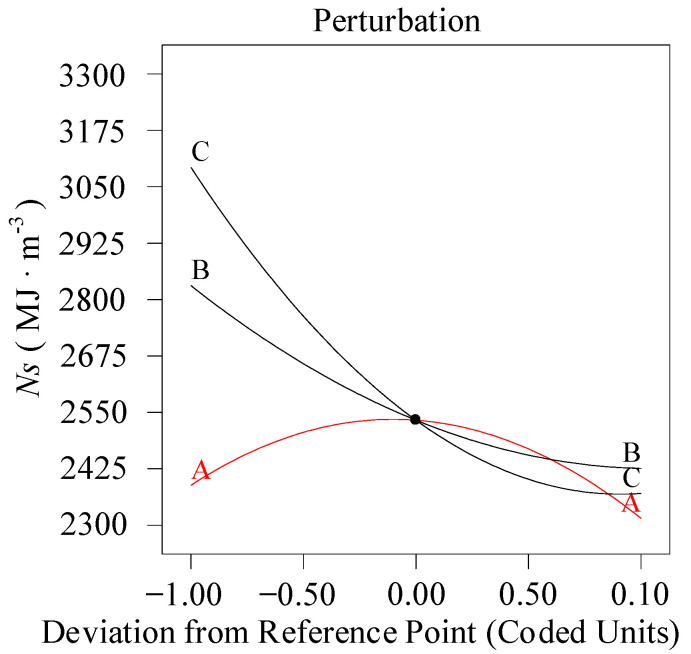
Perturbation diagram (*N_s_*).

According to the regression model shown in the Equation (9), the perturbation diagram under the three-factor action (The intersection point value: A: *a_p_* = 1.375 mm, B: *ν_c_* = 150 m/min, C: *f* = 0.1 mm/rev) is plotted as illustrated in Figure 6. As revealed by the analysis, the specific cutting energy diminishes significantly as the feed rate is on the rise, which conforms to the research results obtained by Bagaber and Yusoff [24], that is, the energy consumption bears negative correlation with feed rate. A higher feed rate will reduce the time required to machine the material, thus reducing the energy consumed to perform cutting. The specific cutting energy reduces with increasing cutting speed, which is seemingly contradictory to the fact that a high power is required for high-speed cutting. While in fact, as the study performed by Parida and Maity [32], this result can be interpret that the cutting force decreases with increasing the cutting speed. When the cutting speed increases, the contact time and friction time of the cutting zone are reduced. In the meantime, the surface of workpiece is softened because of the influence of heat, which causes the shear strength of the material to decline, thus reducing the cutting force. In addition, with the cutting speed increasing, the material removal rate increases, which reduces the time required to remove the same material, thereby suppresses actual energy consumption. The correlation between cutting depth in the selected range and cutting energy is insignificant. Therefore, it is necessary absolutely to select higher feed rate and higher cutting speed in order to reduce energy consumption. 

#### 4.2.2. Surface Roughness (*Ra*)

**Figure 7:**
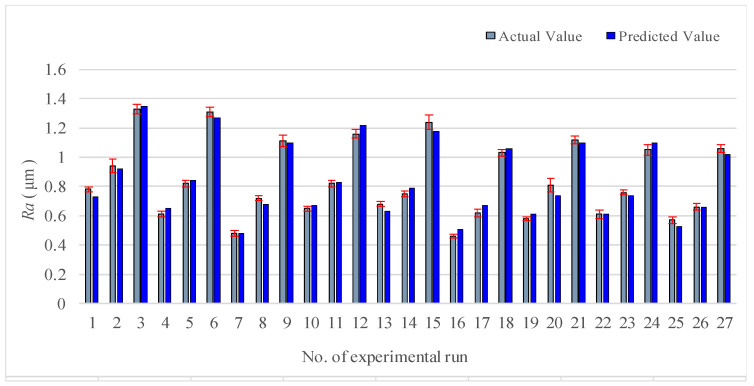
Comparison chart between predicted value and measured value (Ra).

The prediction model of surface roughness is presented in the Equation (10). The value comparison made between the prediction and the measurement is shown in Figure 7. As revealed by the analysis, there is a small error between the two cases and the precision is high.

(10) $$\begin{array}{cc} Ra= & \;0.960\;-0.00040\times v_{c}-1.17\times f-0.239\times a_{p}-0.000012\times \\ & v_{c}{}^{2}+\;45.44\times f^{2}+0.001883\times v_{c}\times a_{p}-1.726\times f\times a_{p}\; \end{array}$$

**Figure 8:**
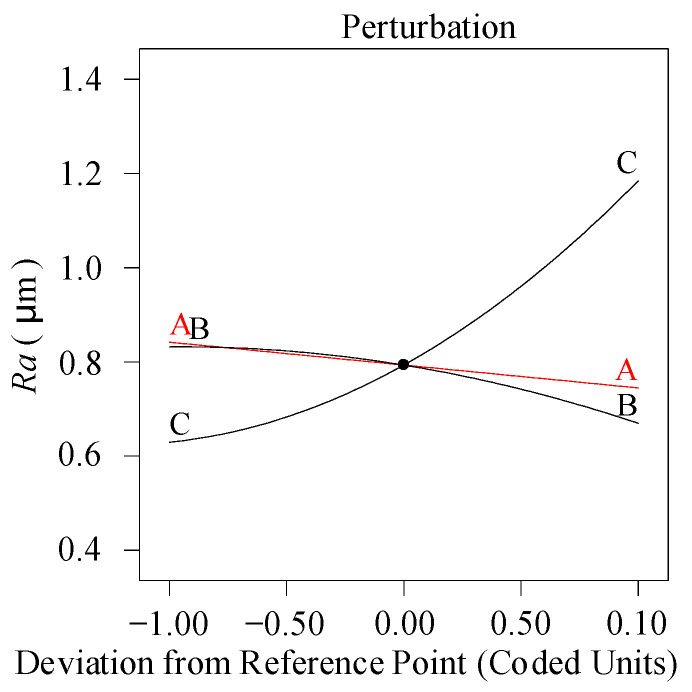
Perturbation diagram (*Ra*).

Figure 8 presents the perturbation diagram under the action of three factors (the intersection point value: A: *a_p_* = 1.375 mm, B: *ν_c_* = 150 m/min, C: *f* = 0.1 mm/r). The figure demonstrates that there is significant positive effect between surface roughness and feed rate. Camposeco-Negrete [11] also reported that the feed rate was the most significant factor influencing surface roughness. Increasing the amount of feed rate will enlarge the height of the remaining cutting zone, which is the main reason for the unevenness of the processed surface. As the cutting speed is on the rise, the surface roughness declines slightly, which is speculated to be caused by the rise of cutting temperature due to the increase in cutting speed, then the material is softened and surface roughness becomes lower. Suresh et al. [12] reached the similar conclusion. In addition, at low or medium speed, scale thorn or built-up edges may appear on the machined surface, which also cause the roughness to deteriorate. The effect of cutting depth variation within the scope of research on surface roughness is insignificant. Therefore, the measure to obtain superior surface roughness is to maintain a high cutting speed and a low feed speed.

#### 4.2.3. Microhardness (*HV*) 

**Figure 9:**
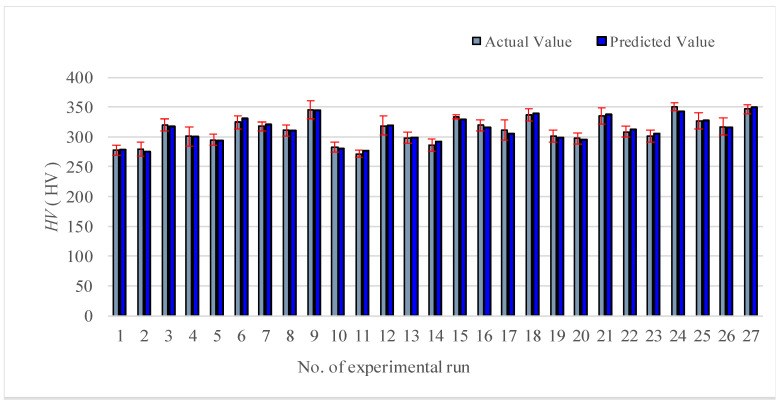
Comparison chart between predicted value and measured value (*HV*).

The regression prediction model of microhardness is presented in the Equation (11). As revealed by the comparison between the prediction and the measurement indicated in Figure 9, the model shows high accuracy, for which the regression model of microhardness is available.

(11) $$\begin{array}{cc} HV\;= & \;347.2-112.5\times a_{p}+0.5689\times v_{c}-1298\times f+55.1\times \\ & a_{p}{}^{2}+9017\times f^{2}-0.1509\times a_{p}\times v_{c}-1.325\times v_{c}\times f \end{array}$$

**Figure 10:**
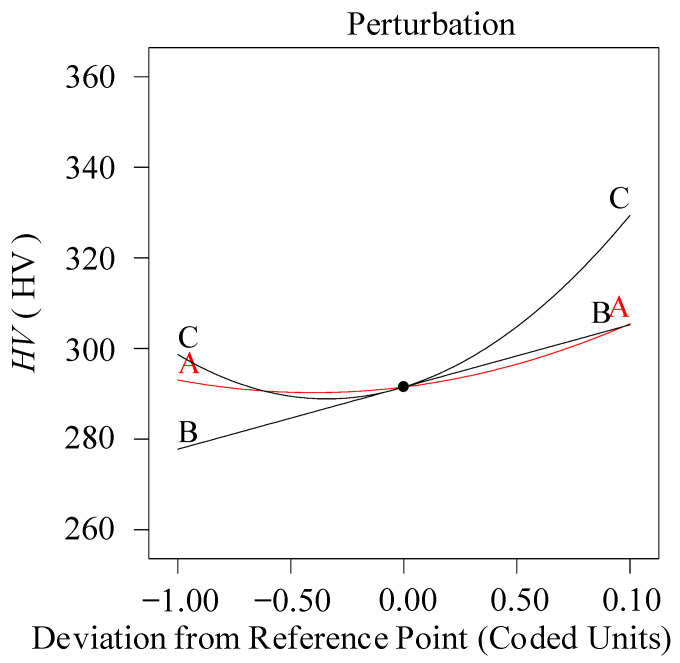
Perturbation diagram (*HV*).

Figure 10 is the perturbation diagram of microhardness (the intersection point value: A: *a_p_* = 1.375 mm, B: *ν_c_* = 150 m/min, C: *f* = 0.1 mm/rev). The analysis demonstrates that the feed rate and cutting speed are the major influencing parameters.

The microhardness value increases as the cutting speed rises, which is the joint result of the reinforcing effect caused by the cutting force and plastic deformation, and the weakening effect caused by the cutting thermal softening. The higher the cutting speed, the smaller the cutting force, and the greater the plastic deformation speed (probably due to the increased strain rate), which results in the hardening enhancement effect overwhelming the reduction effect of the cutting thermal softening. This ends up with the increase in microhardness. As indicated by Cai et al. [33], with cutting speed increasing, strong mechanical loads will impact on the machined surface to cause more significant plastic deformation, which is possibly attributed to the increased strain rate. As a result, the hardening enhancement effect outweighs the reduction influence of the cutting thermal softening. Ultimately, microhardness increases.

**Figure 11:**
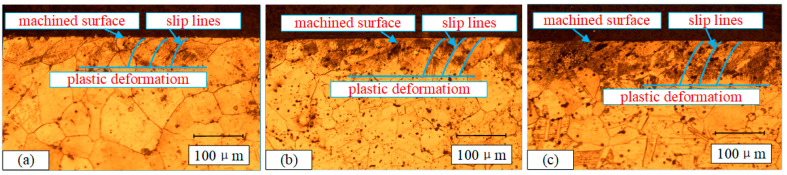
Microstructure micrograph close to the surface layer at different cutting speeds. (**a**) sample 2, (**b**) sample 5, (**c**) sample 8.

With the combined effect of mechanical force and thermal load during the cutting process, the workpiece subsurface microstructure transforms. Figure 11 illustrates the microstructure micrograph close to the surface layer at different cutting speeds. In order to characterize the plastic deformation clearly, Figure 11a–c shows respectively several asymptote lines relative to the cutting speed direction, which represent the deflection angle of grains along the cutting speed direction. The higher cutting speed will lead to more pronounced grain deformation and slip, and this phenomenon becomes more obvious close to the processed surface. This is mainly the effect of extrusion on the mechanical load of the machined surface, and the thermal load caused by the heat generated during the cutting process being transferred to the machined surface.

**Figure 12:**
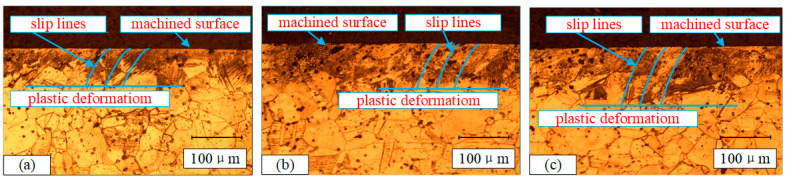
Microstructure micrograph close to the surface layer at different feed rates: (**a**) sample 7; (**b**) sample 8; (**c**) sample 9.

As the feed rate rises, the microhardness declines prior to increase. The reason is that when the feed is relatively large, if the feed rate continues the upward trend, the cutting force rises, and the plastic deformation to the surface layer metal increases, which results in increasing hardening. When the feed rate is relatively low, the main effect is the mechanical load caused by extrusion, which enhances the microhardness more than the thermal softening effect, so the hardening phenomenon will be more obvious. Figure 12 illustrates the microstructure micrograph close to the surface layer at different feed rate. Compared with the feed rate less than 0.1 mm/rev, when it is increased from 0.1 to 0.15 mm/rev, the plastic deformation depth increases significantly. This result shows a high consistency with the microhardness test results.

The effect created by cutting depth on microhardness is less significant in the selected range, as cutting depth has less influence on cutting force and temperature, and plastic deformation per unit area, which is similar to the conclusion drawn by Pawade et al. [34]. Therefore, a higher cutting speed and feed rate ought to be selected to achieve a large microhardness.

**Figure 13:**
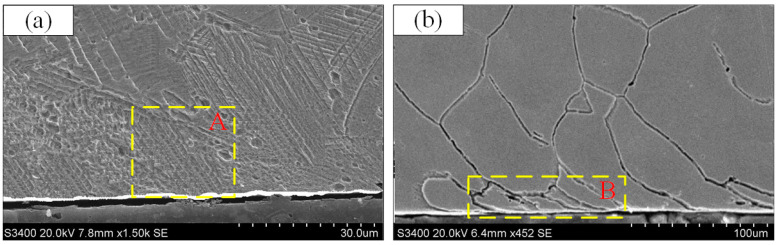
SEM image close to the machined surface for sample 9: (**a**) area A; (**b**) area B.

In order to explain the effect of the cutting process in detail, sample 9 is selected for scanning electron microscope (SEM) experiment. Figure 13 shows the microstructure of the processed surface metamorphic layer. Figure 13a demonstrates obvious slip zones in the metamorphic layer of the area A, and the angle of the slip line in the machined surface is relatively consistent. As the depth from the machined surface increases, the number of slip lines decreases and the slip phenomenon diminishes. Figure 13b indicates that there is obvious grain refinement or damage near surface of the area B. As the temperature of the workpiece rises, a more obvious plastic deformation layer is formed near the machined surface, where the dislocation density increases, the grains are refined, and the material surface is hardened, while the grains in the area below the plastic deformation layer are coarser [35]. In addition, in Figure 13a,b, the white layer can be observed clearly on the surface of the workpiece. The thickness of the white layer is no greater than 10 μm, and the thickness of each section varies. The appearance of the white layer may be due to the severe plastic deformation and phase transformation of the cutting surface layer when the cutting speed is high. At the same time, the appearance of the white layer also indicates the increase in the microhardness of the machined surface layer. This conclusion is consistent with the report of Ding et al. [36].

### 4.3. Multi-Objective Optimization

Desirability analysis is conducted to select the optimal cutting conditions for multi-objective optimization. In Table 9, the constraints on optimization are indicated. In the desirability function, the weight determines the distribution of desirability in the interval between the lower limit (or upper limit) and the goal. A weight of 1 is considered as a neutral setting. The response approaches the goal faster with the increase of weight, while the effect of reducing the weight is just the opposite. All lower weight and upper weight values are set to 1 for eliminating the impact of different optimizing speeds on the results. The importance represents the relative importance of each output response, and the importance value is set according to the state of the objective responses. The ranges of input cutting parameters are set in accordance with the manufacturer’s recommendation. The output objective setting is minimized specific cutting energy and minimized surface roughness, and their value ranges are determined by the maximum and minimum of the experiment. In addition, as a main indicator of surface quality in cutting processing, microhardness is of great significance for evaluating the mechanical properties of the machined surfaces. The increased microhardness is conducive to reducing the wear rate of parts [37], and the microhardness bears a significant positive correlation with the yield strength [27]. Therefore, it is necessary to select a higher level of microhardness range to improve both service life and yield strength. The value of the microhardness is determined as 320–350 HV in combination with microhardness measurement of the 304 stainless steel. As a high cutting speed is beneficial for reducing both specific cutting energy and surface roughness, as well as maintaining a high microhardness, the cutting speed goal is “maximized,” while the goal of feed rate and cutting depth are set to “is in range.” In the process of machining, it can be classed into low energy consumption mode and low surface roughness mode by setting different importance values.

#### 4.3.1. Low Energy Consumption Mode (LEC Mode)

**Figure 14:**
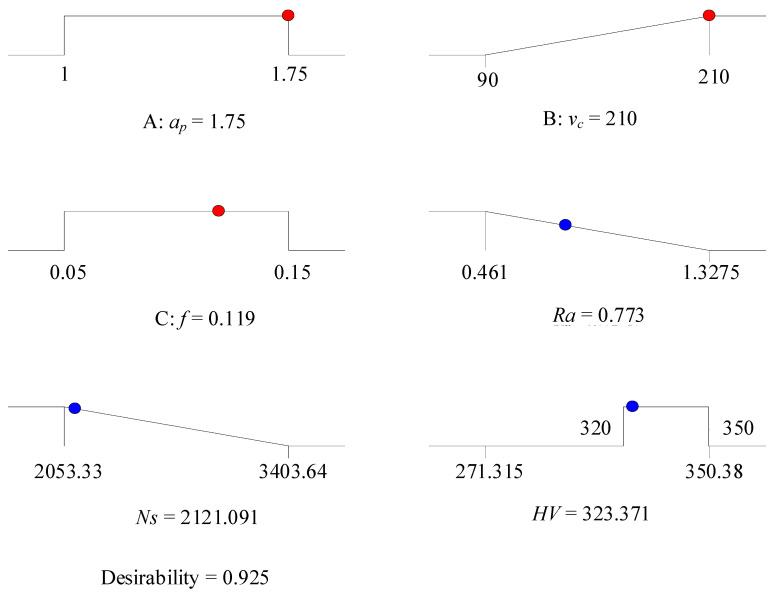
Ramp function graph of desirability (LEC Mode).

**Figure 15:**
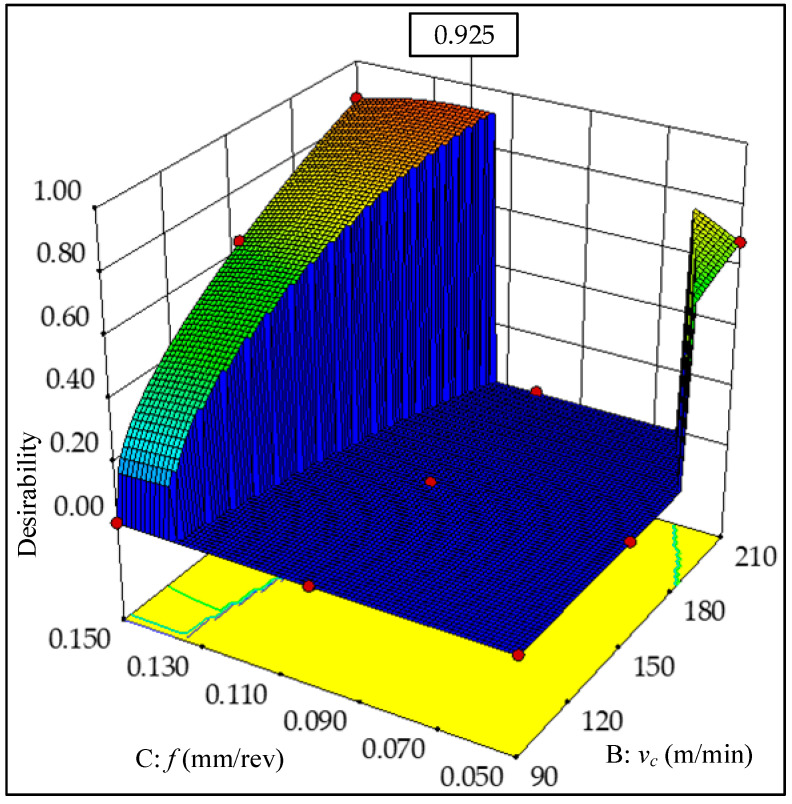
3D surface plots of composite desirability (LEC mode).

**Figure 16:**
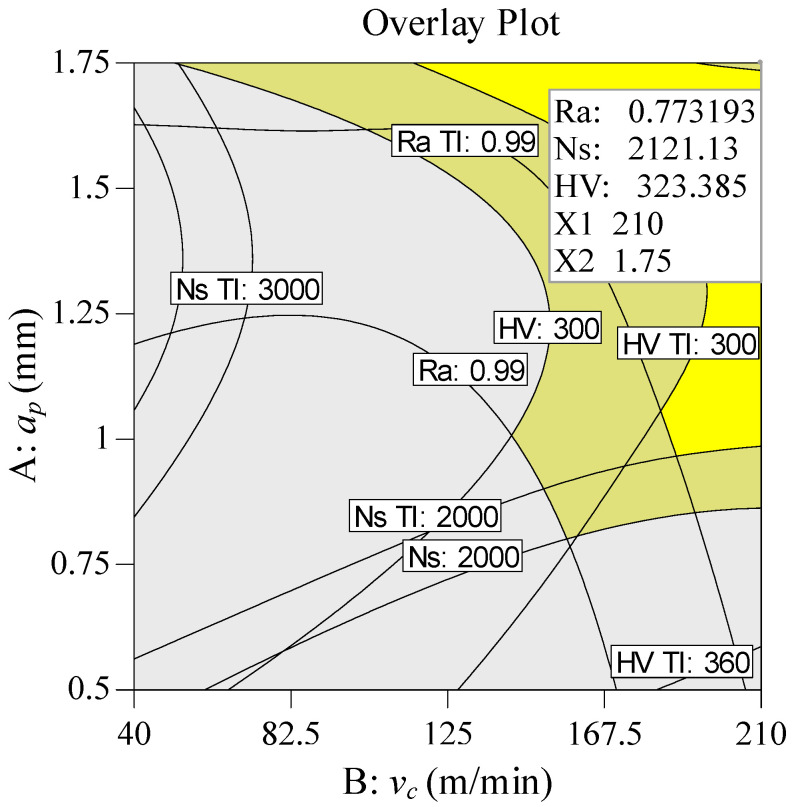
Overlay contour plot for multi-response (LEC mode).

To minimize energy consumption, importance values with high differences are set between specific cutting energy and surface roughness. The importance value of specific cutting energy is *r*_2_ = 5 (+++++), and the importance values of surface roughness are taken as *r*_1_ = 1 (+), *r*_1_ = 2 (++), *r*_1_ = 3 (+++) respectively. The importance values of the remaining variables are set to 3. Table 10 indicates the multi-objective optimization solution that provides a total of five sets of alternative results. As the desirability value is closer to 1, the solution is more desirable. The cutting parameter conditions and corresponding combinations of the solution 1 are selected in three cases. When the value of *r_1_* is different, the cutting depth and cutting speed are unchanged basically, and the feed rate varies in a small range. In order to obtain lower energy consumption, *r*_1_ =1 is taken as an ideal choice. Figure 14 presents the ramp function graph of desirability. The relatively highest composite desirability value (0.925) is obtained, and the optimal combination of operating conditions is *ν_c_* = 210 m/min, *f* = 0.119 mm/rev, *a_p_* = 1.750 mm. In Figure 15, a 3D surface plot of composite desirability is illustrated, which can be referenced to analyze the global situation of composite desirability under various cutting parameter combinations in the mode of low energy consumption. Figure 15 demonstrates that a high value of composite desirability is obtained when the cutting speed is high and the feed rate falls within the range of 0.11–0.15 mm/rev. However, if the feed rate is in the range of 0.07–0.11 mm/rev, the composite desirability value is less than 0.2 regardless of however the cutting speed changes. In order to reflect the feasible range of different responses more intuitively with multiple influencing factors considered, the overlay contour plot is used to analyze the feasibility of the responses. Figure 16 presents the overlay contour plot of three objective responses, cutting speed, and feed rate. The yellow area in Figure 16 denotes the corresponding feasible domain, which can provide a better guidance on the selection of feasible cutting parameters.

#### 4.3.2. Low Surface Roughness Mode (LSR Mode)

**Figure 17:**
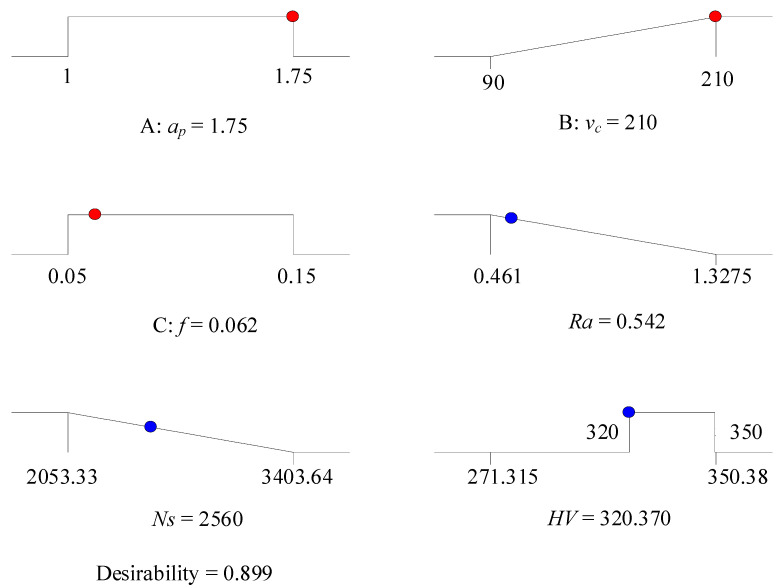
Ramp function graph of desirability (LSR mode).

**Figure 18:**
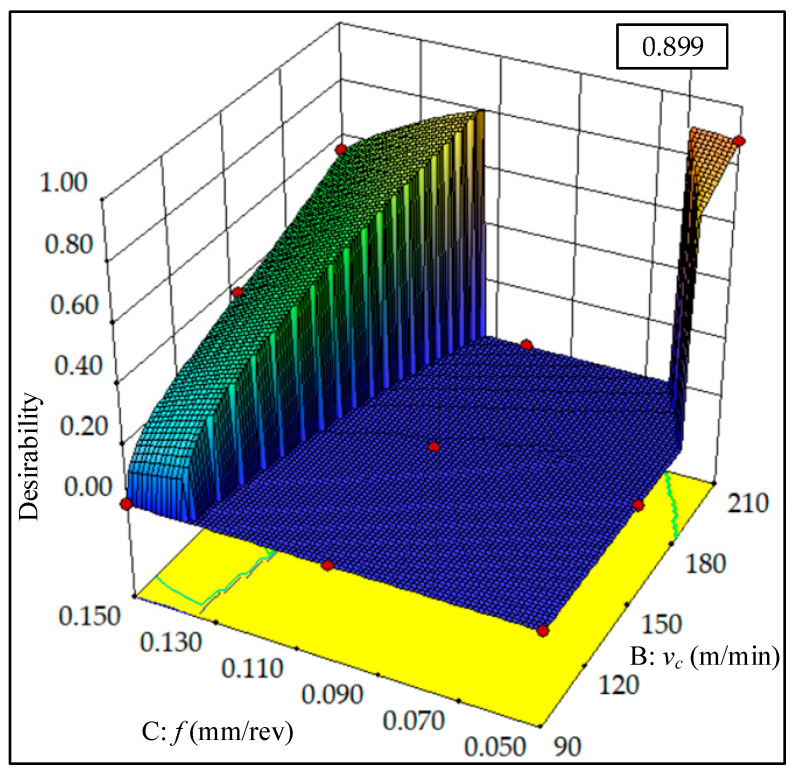
3D surface plots of composite desirability (LSR mode).

**Figure 19:**
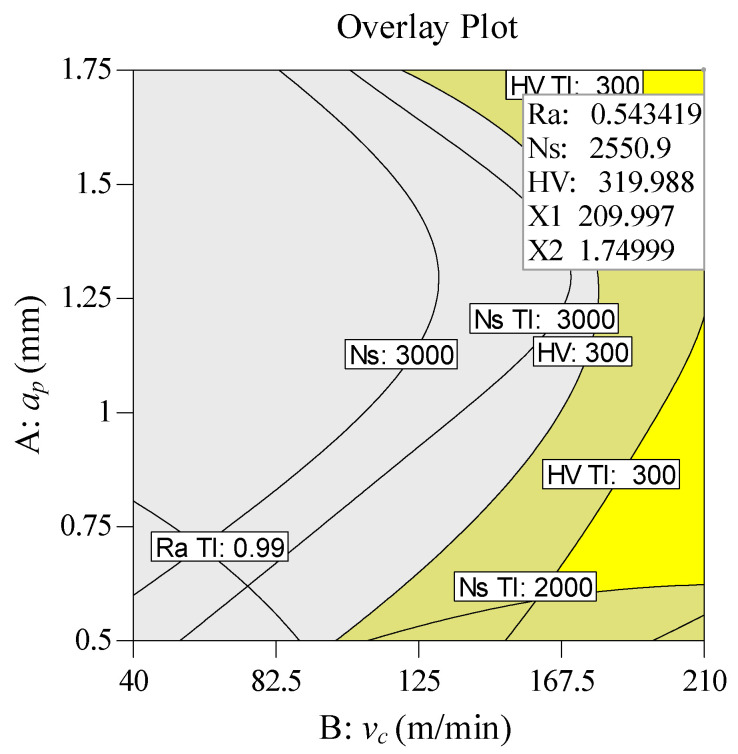
Overlay contour plot for multi-response (LSR mode).

In order to improve the surface quality, set a higher surface roughness importance value *r_1_* = 5(+++++), while the importance values of specific cutting energy are *r*_2_ =1 (+), *r*_2_ = 2 (++), *r*_2_ = 3 (+++), respectively. The importance values of the remaining variables are set to 3. In Table 11, the solution providing five sets of alternative results is indicated. Since the cutting parameters and target values are basically the same, solution 1 with the highest composite desirability (0.899) is selected to achieve the optimum combination of cutting parameters (*ν_c_* = 210 m/min, *f* = 0.062 mm/rev, *a_p_* = 1.750 mm). In Figure 17, the numerical optimization ramps graph of desirability is presented. By conducting an analysis of the 3D surface plot of composite desirability shown in Figure 18, the global distribution of the composite desirability values can be determined under the influence of different cutting parameters. Figure 19 illustrates the overlay contour plot of low surface roughness mode, where the yellow areas indicate the feasible domains for the three objective responses.

### 4.4. Optimization Result Verification

#### 4.4.1. Genetic Algorithm Optimization

##### Optimization by MOGA

**Figure 20:**
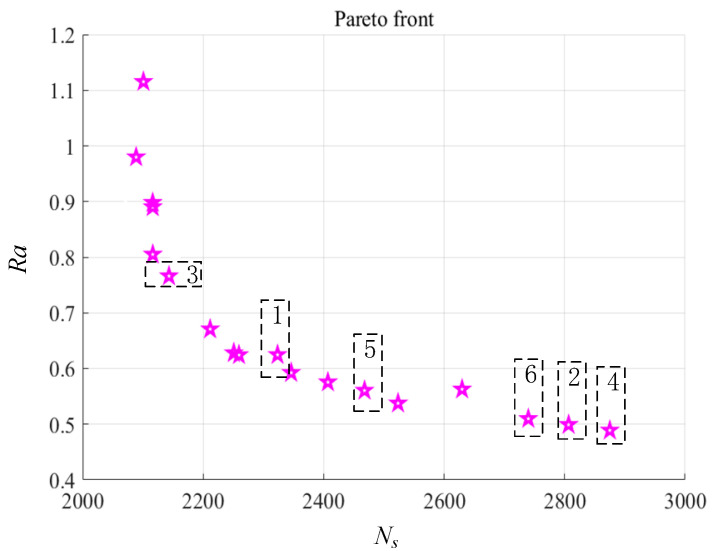
Pareto frontier obtained by multi-objective genetic algorithm (MOGA).

According to the constraints on optimization in Table 9, the optimal parameters for the minimum specific cutting energy and minimum surface roughness are determined by the multi-objective genetic algorithm (MOGA). The specific cutting energy model in Equation (9) and the surface roughness model in Equation (10) are selected as optimization goals. Several experiments are carried out and the Pareto frontier curve is obtained as shown in Figure 20. Table 12 indicates six sets of Pareto frontier points of the goals, which are marked correspondingly in Figure 20. Pareto front points obtained by MOGA is a set of solutions after weighing the two responses and the values are different. They are potentially the optimal solution to optimization, but it is difficult to accurately determine the solution needed. The results show that the third and fifth groups in the Pareto solution set have similar parameters combinations with LEC mode and LSR mode respectively.

##### Optimization by GA Combined with Weighted-Sum-Type Objective Function

**Figure 21:**
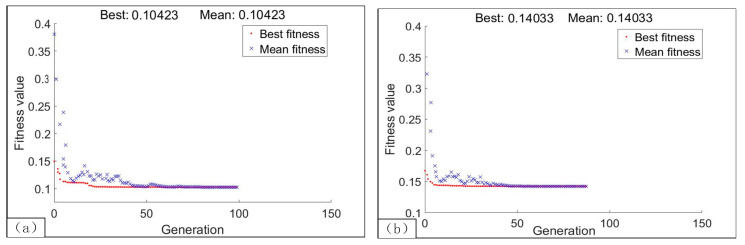
Fitness function values by genetic algorithm (GA): (**a**) LEC mode; (**b**) LSR mode.

**Figure 22:**
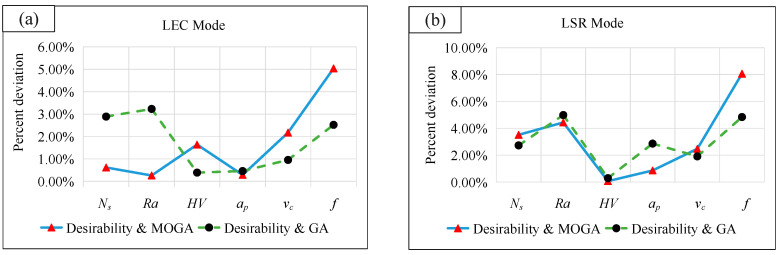
Deviation percentage analysis: (**a**) LEC mode; (**b**) LSR mode.

The multi-objective problem can be transformed into single-objective optimization by weighted-sum-type objective function, and then the genetic algorithm (GA) is used to search the single optimal solution [38]. The above optimization constraints and goals are still used. The two objective response models are normalized, weighted, and summed to obtain the single objective function in Equation (12). Then the GA algorithm is used for optimization.

(12) $$y=w_{1}\times \frac{N_{s}-N_{s}\left(\min\right)}{N_{s}(\max)-N_{s}\left(\min\right)}+w_{2}\times \frac{Ra-Ra\left(\min\right)}{Ra\left(\max\right)-Ra\left(\min\right)}$$

where *w*_1_ and *w*_2_ represent the weighting factors of the specific cutting energy and surface roughness respectively, *N_s_* and *Ra* denote the values of the two responses obtained by the models, *N_s_*(max) and *N_s_*(min) represent the maximum and minimum values of the specific cutting energy within the parameter range, *Ra*(max) and *Ra*(min) are the maximum and minimum values of surface roughness within the parameter range respectively. In order to be consistent with desirability multi-objective optimization, the ratio of *w_1_* and *w_2_* is between 3:1 and 5:1 for the LEC mode. While for the LSR mode, the corresponding value is determined between 1:5 and 1:3. Figure 21 reports the fitness function values and Table 13 shows the optimization results. The percent deviation obtained by comparing with different methods is shown in Figure 22.

The results show that the combination of cutting parameters obtained by the desirability method and the other two algorithms are close, exhibiting a maximum deviation of 8.06%. The maximum deviation of the objective response is 4.98%, so all numerical deviations are within 10%, indicating that the desirability method is feasible. In addition, despite intelligent algorithms have high accuracy, while they still rely on a mass of data sets. Moreover, the optimization by MOGA is incapable to determine the required solution accurately, and the optimization by GA combined with weighted-sum-type objective function requires multiple times of iterative debugging. In contrast, the desirability method shows such advantages as a higher fitting speed and the ease of operation when small sample data are optimized, which makes it more suitable for the optimization process in this study.

#### 4.4.2. Experimental Verification

The experimental verification is conducted to further illustrate the reliability of the optimization results. With respect to the optimal cutting parameter combination under low energy consumption (I) *ν_c_* = 210 m/min, *f* = 0.119 mm/rev, *a_p_* = 1.750 mm and optimal cutting parameter combination under low surface roughness (II) *ν_c_* = 210 m/min, *f* = 0.062 mm/rev, *a_p_* = 1.750 mm, the cutting experiment is conducted respectively, and the results obtained from confirmation test for responses are indicated in Table 14. As revealed by comparative analysis, the deviation between the multi-objective optimization result and the experimental result is within 7% for the specific cutting energy, surface roughness, and microhardness in the two modes, which suggests that the optimization result of this study is acceptable.

**Figure 23:**
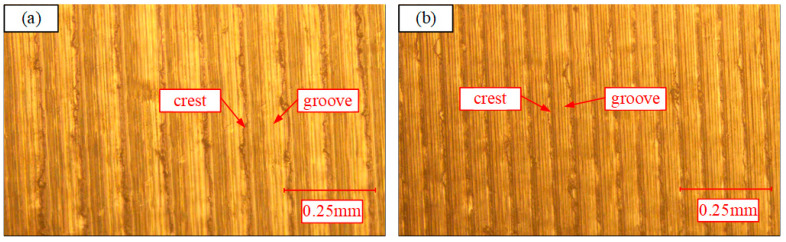
Microscopic images of the surface: (**a**) LEC mode; (**b**) LSR mode.

Figure 23a,b illustrates the microscopic images of the surface for low energy consumption mode (I) and low surface roughness mode (II) under the action of optical microscope respectively. As can be noticed from Figure 23, when the feed rate increases, the surface grooves become deep and rough significantly and the crests distance becomes larger, so the surface roughness of the workpiece becomes worse. It is consistent with the previous analysis results.

## 5. Conclusions

In this study, the regression models of specific cutting energy, surface roughness, and microhardness are constructed separately by the analysis of variance (ANOVA) during the turning of 304 stainless steel, and the influence mechanism between cutting parameters and three output responses are explored. Then, multi-objective optimizations are carried out using the desirability analysis method in two different modes. The major conclusions are drawn as follows:

1. The multiple correlation coefficients (R-Sq) of the three regression models are 98.24%, 97.81%, and 97.31% respectively. Therefore, the developed models are reliable and beneficial for making accurate predictions in machining.
2. It is demonstrated that for specific cutting energy the feed rate is the most significant influencing factor while the correlation of cutting depth is less obvious. A higher cutting speed (210 m/min) and feed rate (0.15 mm/rev) reduce the specific cutting energy, owing to the increase of material removal rate and thermal softening effect.
3. A better surface roughness can be achieved at a lower feed rate (0.05 mm/rev) rather than a higher feed rate (0.15 mm/rev). The reason is that the height of the remaining cutting zone increases with increase in the feed rate. Besides, as the cutting speed rises, the value of surface roughness declines slightly.
4. The microhardness declines before increase as the feed rate rises, which results from a combination of the reinforcing effect caused by cutting force and plastic deformation, and the weakening effect of thermal softening. With the increase of cutting speed, grain deformation and slip become significant. At the same time, microhardness increases.
5. At a high microhardness range of 320–350 HV, the desirability analysis method is applied for multi-objective optimization. The optimum cutting conditions for the LEC mode are as follows: *ν_c_* = 210 m/min, *f* = 0.119 mm/rev, *a_p_* = 1.750 mm. While a set of optimal solutions are determined with the parameter combinations of *ν_c_* = 210 m/min, *f* = 0.062 mm/rev, *a_p_* =1.750 mm.
6. The composite desirability values of optimal solutions in the two modes are 0.925 and 0.899 respectively. Compared with the results of two intelligent algorithms and experiment, the deviation is within 10%, suggesting that the desirability analysis results are satisfactory for optimization. In addition, future research will involve optimization of various sustainable parameters for different types of lubrication conditions.

## Figures and Tables

**Table 1:** Composition of workpiece material.

Element	Si	Mn	P	S	Ni	Cr	C	Fe
Mass Fraction (%)	0.75	1.64	0.045	0.03	8.56	18.87	0.08	70.025 (Balance)

**Table 2:** Geometric and working angels.

Angle	Tool Angle (*ε_r_*)	Rake Angle (*γ_0_*)	Clearance Angle (*α_0_*)	Main Cutting Edge Angle (*K_r_*)	End Cutting Edge Angle (*K^’^_r_*)	Inclination Angle (*λ_s_*)	Approach Angle (*ψ_r_*)
Value (°)	80	8	7	95	−5	−5	−5

**Table 3:** Experimental factors and levels.

	Level 1	Level 2	Level 3
**Process Parameters**	−1	0	1
Cutting Speed: *ν_c_* (m/min)	90	150	210
Feed Rate: *f* (mm/rev)	0.05	0.10	0.15
Cutting Depth: *a_p_* (mm)	1.000	1.375	1.750

**Table 4:** Design and results of the experiment.

No.	*ν_c_* (m/min)	*f* (mm/rev)	*a_p_* (mm)	Experiment Result			Calculation Results
				*F_c_* (N)	*Ra* (μm)	*HV* (HV)	*N_s_* (MJ/m^3^)
1	−1	−1	−1	167	0.78	278.55	3340.00
2	−1	0	−1	272	0.94	279.28	2720.00
3	−1	1	−1	372	1.33	319.745	2480.00
4	0	−1	−1	150	0.61	300.94	3000.00
5	0	0	−1	236	0.82	295.428	2360.00
6	0	1	−1	330	1.31	324.768	2200.00
7	1	−1	−1	144	0.48	317.995	2880.00
8	1	0	−1	223	0.72	311.023	2230.00
9	1	1	−1	308	1.11	345.018	2053.33
10	−1	−1	0	234	0.65	282.528	3403.64
11	−1	0	0	374	0.82	271.315	2720.00
12	−1	1	0	534	1.16	318.915	2589.09
13	0	−1	0	214	0.68	298.67	3112.73
14	0	0	0	360	0.75	286.02	2618.18
15	0	1	0	501	1.24	333.558	2429.09
16	1	−1	0	205	0.46	319.147	2981.82
17	1	0	0	334	0.62	311.617	2429.09
18	1	1	0	470	1.03	337	2278.79
19	−1	−1	1	278	0.58	301.28	3177.14
20	−1	0	1	467	0.81	297.47	2668.57
21	−1	1	1	653	1.12	335.605	2487.62
22	0	−1	1	243	0.61	308.8	2777.14
23	0	0	1	395	0.76	300.79	2257.14
24	0	1	1	564	1.06	350.38	2148.57
25	1	−1	1	232	0.57	326.807	2651.43
26	1	0	1	399	0.66	317.103	2280.00
27	1	1	1	566	1.06	346.153	2156.19

**Table 5:** Correlation between factors and responses.

	*ν_c_*	*f*	*a_p_*	*R_a_*	*N_s_*
*f*	0				
*a_p_*	0	0			
*R_a_*	−0.264	0.901	−0.158		
*N_s_*	−0.447	−0.796	−0.081	−0.557	
*HV*	0.523	0.585	0.236	0.446	−0.634

**Table 6:** ANOVA and R-squared for specific cutting energy (*N_s_*).

Source	DF	Sum of Squares	Mean Square	F Value	*p*-Value	Remark
Model	7	3,637,136	519,591	151.47	<0.0001	Significant
*ν_c_*	1	738,279	738,279	215.22	<0.0001	
*f*	1	2,348,103	2,348,103	684.5	<0.0001	
*a_p_*	1	24,166	24,166	7.04	0.016	
*ν_c_^2^*	1	54,850	54,850	15.99	0.0008	
*f* *^2^*	1	237,423	237,423	69.21	<0.0001	
*a_p_^2^*	1	196,533	196,533	57.29	<0.0001	
*f* × *a_p_*	1	37,782	37,782	11.01	0.0036	
Error	19	65,177	3430			
Total	26	3,702,313				
Model Summary		S	R-sq	Adj R-Sq	Pred R-Sq	
		58.5693	98.24%	97.59%	96.54%	

**Table 7:** ANOVA and R-squared for surface roughness (*Ra*).

Source	DF	Sum of Squares	Mean Square	F Value	*p*-Value	Remark
Model	7	1.66768	0.23824	121.09	<0.0001	Significant
*ν_c_*	1	0.1188	0.1188	60.38	<0.0001	
*f*	1	1.38414	1.38414	703.53	<0.0001	
*a_p_*	1	0.04238	0.04238	21.54	<0.0001	
*ν_c_^2^*	1	0.01083	0.01083	5.51	0.030	
*f* *^2^*	1	0.07744	0.07744	39.36	<0.0001	
*ν_c_ × a_p_*	1	0.02153	0.02153	10.94	0.004	
*f* × *a_p_*	1	0.01256	0.01256	6.39	0.021	
Error	19	0.03738	0.00197			
Total	26	1.70506				
Model Summary		S	R-sq	Adj R-Sq	Pred R-Sq	
		0.04436	97.81%	97.00%	95.63%	

**Table 8:** ANOVA and R-squared for microhardness (*HV*).

Source	DF	Sum of Squares	Mean Square	F Value	*p*-Value	Remark
Model	7	12,069.5	1724.21	98.12	<0.0001	Significant
*ν_c_*	1	3394.22	3394.22	193.16	<0.0001	
*f*	1	4245.02	4245.02	241.58	<0.0001	
*a_p_*	1	692.44	692.44	39.41		
*f* *^2^*	1	3049.04	3049.04	173.52	<0.0001	
*a_p_^2^*	1	360.87	360.87	20.54	<0.0001	
*ν_c_ × a_p_*	1	138.40	138.40	7.88	0.011	
*f* × *a_p_*	1	189.49	189.49	10.78	0.004	
Error	19	333.9	17.57			
Total	26	12,403.3				
Model Summary		S	R-sq	Adj R-Sq	Pred R-Sq	
		4.19186	97.31%	96.32%	95.18%	

**Table 9:** Constraints on multi-objective optimization.

Name	Goal	Lower Limit	Upper Limit	Lower Weight	Upper Weight	Importance
*a_p_* (mm)	is in range	1	1.75	1	1	3
*ν_c_* (m/min)	maximize	90	210	1	1	3
*f* (mm/rev)	is in range	0.05	0.15	1	1	3
*Ra* (μm)	minimize	0.461	1.3275	1	1	*r_1_*
*N_s_* (MJ/m^3^)	minimize	2053.33	3403.64	1	1	*r_2_*
*HV* (HV)	is in range	320	350	1	1	3

**Table 10:** Multi-objective optimization solution (low energy consumption (LEC) mode).

**No. (*r*_1_ = 1)**	***a_p_***	***ν_c_***	***f***	***Ra***	***N_s_***	***HV***	**Desirability**	
1	1.750	210.000	0.119	0.773	2121.091	323.371	0.925	***Selected***
2	1.750	210.000	0.118	0.771	2122.868	323.050	0.925	
3	1.750	210.000	0.118	0.766	2124.548	322.760	0.925	
4	1.750	210.000	0.122	0.790	2113.650	324.908	0.924	
5	1.750	210.000	0.116	0.756	2129.858	321.916	0.924	
**No. (*r*_1_ = 2)**	***a_p_***	***ν_c_***	***f***	***Ra***	***N_s_***	***HV***	**Desirability**	
1	1.750	210.000	0.112	0.731	2144.881	320.001	0.896	***Selected***
2	1.750	209.914	0.113	0.735	2142.611	320.243	0.896	
3	1.750	210.000	0.115	0.748	2134.013	321.326	0.895	
4	1.750	209.648	0.114	0.741	2138.639	320.680	0.894	
5	1.750	210.000	0.116	0.754	2130.764	321.783	0.894	
**No. (*r*_1_ = 3)**	***a_p_***	***ν_c_***	***f***	***Ra***	***N_s_***	***HV***	**Desirability**	
1	1.750	209.999	0.112	0.731	2144.885	320.000	0.875	***Selected***
2	1.750	209.992	0.113	0.734	2143.231	320.183	0.874	
3	1.747	210.000	0.113	0.735	2145.165	320.146	0.873	
4	1.750	210.000	0.114	0.740	2139.355	320.650	0.873	
5	1.750	210.000	0.115	0.747	2135.201	321.185	0.872	

**Table 11:** Multi-objective optimization solution (low surface roughness (LSR) mode).

**No. (*r*_2_ = 1)**	***a_p_***	***ν_c_***	***f***	***Ra***	***N_s_***	***HV***	**Desirability**	
1	1.750	210.000	0.062	0.542	2560.460	320.370	0.899	***Selected***
2	1.745	210.000	0.062	0.542	2561.801	320.002	0.898	
3	1.750	210.000	0.056	0.536	2625.128	322.992	0.894	
4	1.718	210.000	0.059	0.537	2627.012	320.000	0.893	
5	1.750	209.999	0.054	0.535	2651.136	324.127	0.892	
**No. (*r*_2_ = 2)**	***a_p_***	***ν_c_***	***f***	***Ra***	***N_s_***	***HV***	**Desirability**	
1	1.750	209.996	0.062	0.543	2550.721	320.002	0.868	***Selected***
2	1.746	210.000	0.062	0.543	2559.931	320.000	0.866	
3	1.750	210.000	0.061	0.542	2566.589	320.579	0.866	
4	1.750	209.103	0.062	0.545	2556.147	320.000	0.864	
5	1.750	209.999	0.059	0.539	2592.417	321.628	0.862	
**No. (*r*_2_ = 3)**	***a_p_***	***ν_c_***	***f***	***Ra***	***N_s_***	***HV***	**Desirability**	
1	1.750	209.996	0.062	0.543	2550.721	320.002	0.868	***Selected***
2	1.746	210.000	0.062	0.543	2559.931	320.000	0.866	
3	1.750	210.000	0.061	0.542	2566.589	320.579	0.866	
4	1.750	209.103	0.062	0.545	2556.147	320.000	0.864	
5	1.750	209.999	0.059	0.539	2592.417	321.628	0.862	

**Table 12:** Partial Pareto front points by MOGA.

Index	*N_s_*	*Ra*	*HV*	*a_p_*	*ν_c_*	*f*
1	2315.121	0.625	320.124	1.739	206.269	0.098
2	2810.223	0.506	318.512	1.221	201.654	0.048
3	2134.12	0.771	328.672	1.745	205.434	0.125
4	2875.559	0.486	318.122	1.021	201.34	0.051
5	2470.509	0.566	320.568	1.735	204.815	0.067
6	2745.224	0.515	321.089	1.718	201.681	0.053

**Table 13:** Optimization results through GA.

	Results	
**Optimal Responses**	**LEC Mode**	**LSR Mode**
*N_s_*	2182.387	2630.304
*Ra*	0.798	0.515
*HV*	322.12	321.30
**Optimal Cutting Parameters**		
*a_p_*	1.742	1.700
*ν_c_*	212	206
*f*	0.116	0.059

**Table 14:** Results of confirmation experiment for responses.

**Parameters (I)**		**Optimum Value**			**Experimental Value**		
		***Ra***	***N_s_***	***HV***	***Ra***	***N_s_***	***HV***
*a_p_*	1.750	0.773	2121.091	323.371	0.791	2199.213	306.127
*ν_c_*	210.0						
*f*	0.119						
Error					0.018	78.122	17.244
Proportion					2.28%	3.55%	5.63%
**Parameters (II)**		**Optimum Value**			**Experimental Value**		
		***Ra***	***N_s_***	***HV***	***Ra***	***N_s_***	***HV***
*a_p_*	1.750	0.542	2560.460	320.370	0.557	2409.167	331.102
*ν_c_*	210.0						
*f*	0.062						
Error					0.015	151.293	10.732
Proportion					2.69%	6.28%	3.24%
